# Community ecological response to polycyclic aromatic hydrocarbons in Baiyangdian Lake based on an ecological model

**DOI:** 10.1007/s10646-023-02722-y

**Published:** 2024-01-06

**Authors:** Yong Zeng, Jiaxin Li, Yanwei Zhao, Wei Yang

**Affiliations:** 1 State Key Laboratory of Heavy Oil Processing, Beijing Key Laboratory of Oil & Gas Pollution Control, College of Chemical Engineering and Environment, China University of Petroleum, Beijing, 102249 China; 2 State Key Laboratory of Water Environment Simulation, School of Environment, Beijing Normal University, Beijing, 100875 China

**Keywords:** Ecological model, Toxicity, Interaction strength, Polycyclic aromatic hydrocarbons, Response pattern, Baiyangdian Lake

## Abstract

The dynamic response of a single population to chemicals can be represented by a Weibull function. However, it is unclear whether the overall response can still be represented in this manner when scaled up to the community level. In this study, we investigated the responses of biological communities to polycyclic aromatic hydrocarbons by using an ecological model of Baiyangdian Lake in northern China. The community dynamics process was divided into the following three stages. In the first stage, toxicity, played a dominant role and strong, medium, and weak species responses were observed according to the toxicity sensitivity. In the second stage, the dynamic process was dominated by the interaction strength with three alternative dynamic pathways comprising of direct response, no response, or inverse response. In the third stage, the toxicity was again dominant, and the biomasses of all species decreased to extinction. The toxicological dynamics were far more complex at the community level than those at the single species level and they were also influenced by the interaction strength as well as toxicity. The toxicological dynamic process in the community was constantly driven by the competing effects of these two forces. In addition to the total biomass, the interaction strength was identified as a suitable community-level signal because it exhibited good indicator properties regarding ecosystem steady-state transitions. However, we found that food web stability indicators were not suitable for use as community-level signals because they were not sensitive to changes in the ecosystem state. Some ecological management suggestions have been proposed, including medium to long-term monitoring, and reduction of external pollution loads and bioindicators. The results obtained in this study increase our understanding of how chemicals interfere with community dynamics, and the interaction strength and total biomass were identified as useful holistic indicators.

## Introduction

It is well known that the Earth’s biodiversity is declining (McCann, 2000). Pollution is a major threat to the biodiversity of ecosystems worldwide and it can reduce the provision of ecosystem services (Gredelj et al., 2018). Polycyclic aromatic hydrocarbons (PAHs) are persistent organic pollutants and their ecological effects and potential toxicity have been widely reported (Miao et al., 2023; Han et al., 2022). Traditional assessments of ecological effects are based on chemical analysis and biological toxicity tests in a single species exposed to toxicants (Zhang et al., 2018). However, this method has been criticized because of its lack of ecological realism. Real ecosystems are environments under multiple stresses with community dynamics (Lombardo et al., 2015; Grechi et al., 2016). Therefore, new tools need to be developed and applied that consider the complexity of communities and ecosystems (Zhang et al. 2018). Indeed, ecological models have been proposed to overcome these limitations (Galic et al., 2010). In particular, recent ecological models, such as PCLake (Kong et al., 2017) and Aquatox (Zhang et al., 2013, 2014, 2020), have been used to assess the ecological effects of chemicals entering ecosystems. Most studies have focused on the effects of toxic chemical substances or other pollutants on species biomass (Fu et al., 2019), eco-exergy (Jørgensen et al., 2005), and the production–respiration ratio, or on indicators of ecological net analysis (Fath et al. 2019). However, relatively little attention has been paid to the responses of communities to a chemical after it enters ecosystems, or its effects on the structure and stability of a food web.

An ecological community can be viewed as a network of species connected by interspecific interactions called a food web (Karlsson et al., 2007; Mougi & Kondoh, 2012; Kuiper et al., 2015). According to May (1972), the stability of a food web depends on the network size (S), connectance (C), and average interaction strength (*α*). Recent studies have verified community-level parameters that represent species diversity and interactions. These parameters are sufficient to predict the dynamic behavior of complex ecological communities (de Ruiter et al., 1995; Ives & Carpenter, 2007; Bunin, 2017; Hu et al., 2022). Recently, interaction intensity and stability indicators have been applied to evaluate the structure and function of eutrophic ecosystem (Zhang et al., 2022, 2023). Another type of indicator that describes the structure and function of ecosystems is ecological network analysis (ENA) (Christian et al., 2009; de la Vega, et al., 2018, Safi et al., 2019), which uses many indicators. Currently, there is no consensus on a short list to describe the structure and function of an ecosystem (de Jonge & Schückel, 2021). However, few studies have investigated how the toxicity of a chemical affects the interaction strength and stability of a food web.

The concentration–response relationship between the internal concentrations of PAHs and their biological effects in a single species, or variations in the internal concentration over time, can be described using a non-linear Hill-based toxicodynamic model (Chen et al., 2021), where the response at the single species level follows a familiar sigmoid-shaped curve, such as a logistic function or Weibull function (Christensen & Nyholm, 1984; Vázquez, 2011; Gadagkar & Call, 2015). This relationship has been confirmed based on toxicological tests in many species and it has been widely used in species sensitivity distributions (Zheng et al., 2015) and toxicokinetic modeling (Zhu et al., 2022). However, a rule that is suitable for a single species might not work in community environments where interference is caused by the interspecific or intraspecific interaction strength. Thus, a question is how do multiple species in a food web respond to PAHs under the combined effects of toxicity and the interaction strength? Answering this question will improve our understanding of the mechanisms that allow community dynamics to respond to toxic interference.

Polycyclic aromatic compounds have extremely low water solubility and are difficult to eliminate in the environment, so PAHs are identified as priority pollutants by both the US Environmental Protection Agency and the European Community (Han et al., 2021). The total PAHs concentration in the sediments of BYD Lake ranged from 97.2 to 2402 ng g^−1^ dw, much higher than the mean values of Chinese Lakes (478 ± 812 ng g^−1^ dw) (Guo et al., 2011; Xia et al., 2023). In addition, most existing studies for BYD lake have focused on the toxic effects of chemicals (Zhang et al., 2014, 2018, 2020; Zeng et al., 2022), ignoring the underlying mechanisms and community level indicators. It is necessary to develop indicators that reflect the integrity of ecosystems after understanding the community dynamics driven by PAHs.

To address this problem, we used an ecological model to study the changes in the community dynamics in response to PAHs in Baiyangdian (BYD) Lake in northern China. The aims of this study were: (1) to explore the community response of the ecosystem under interference from PAHs in BYD Lake; and (2) to analyze the differences in indicators of the total biomass, interaction strength, and food web stability under PAH concentration gradients.

## Materials and methods

### AQUATOX model of BYD Lake in response to PAHs

AQUATOX 3.1 considers the cause–effect mechanisms between biological responses and chemical toxicity through trophic-level relationships from primary producers to fish (Martins et al., 2019) based on a series of differential equations that describe the mass balance of the species size and toxicodynamic processes. In this study, we used the calibrated BYD Lake AQUATOX model developed by Zeng et al. (2022) to predict the dynamics of the species biomass under different PAHs concentration gradients in BYD Lake. Technical details regarding parameterization of the ecosystem model were given by Zeng et al. (2022). Based on previous biological surveys and relevant literature (Zeng, et al., 2021), the main representative species were selected. The following 12 species were simulated in BYD Lake: two microalgae (blue-green, diatom), one macrophyte, three zooplankton (rotifer, copepod, cladoceran), four benthic animals (chironomid, oligochaeta, mussel, gastropod), and two fishes (carp, catfish). The input parameters of the model mainly include physicochemical parameters, biological parameters, and parameters of PAHs, such as physicochemical properties, toxicokinetic parameters, toxicological parameters, initial concentrations, and loadings. For each modeled species, the initial values of biomass were taken from biomonitoring data in April 2018. The starting values of the kinetic parameters came from previous literature or the default values of the most similar species in the AQUATOX 3.1 library. The time period for the simulations ranged from March 1, 2018 to March 1, 2019, and the simulation time step was one day. The model calibration results indicate that the overall average biomass relative variation (ε) was 17.56% and the average relative variation of each species (εi) was less than 30%. The calibration results are acceptable.

The concentration of total PAHs was expressed as benzo(a)pyrene (BaP) equivalents based on toxicity comparisons (Nisbet & Lagoy, 1993). BaP concentration in water during March, 2018~March, 2019 had been detected with 7.81~35.71 ng/L, average 19.17 ng/L. The ecological effects of BaP equivalents were derived in terms of differences in the biota biomass density between control model runs (i.e., without pollutants) and perturbed model runs (i.e., with pollutants). Based on the calibrated model, the “with-without BaP” simulations were performed twice with the current value (March 1, 2018) as the initial value. The last two months average output values of “without BaP “simulation were considered as the stable state before BaP poisoning. While, last two months average values of “with BaP” simulation were considered as the stable state after BaP poisoning. The period between the two states is the transition stage.

Next, the toxicokinetic process in the AQUATOX model is briefly introduced. As a species mortality item, the biomass of given organisms killed by exposure to a toxicant at a given time is described by the cumulative form of the Weibull distribution with the internal concentration of the toxicant and a shape parameter. The internal concentration of a toxicant is calculated by using mass balance equations for toxicants in organisms, and the shape parameter expresses the variability in the toxic response (unitless). The lethal internal concentration of a toxicant that causes 50% mortality is related to the median lethal concentration (LC_50_), bioconcentration factor, and exposure time. Sublethal effects of chemicals, such as the half-effective concentration (EC_50_), are estimated as a fraction of the lethal effects. Detailed descriptions of the processes and equations used in the model can be found in the technical reference guide for the model (Park & Clough, 2018).

### Ecological response indicators

The ecologists have sought to predict community behaviors with simple community-level parameters such as the number of species and the distribution of interaction strengths between species (Hu et al., 2022). Therefore, the principle of selecting indicators was of course simple and effective. In characterizing the structure and function of ecosystems, the indicators seem to have two types, one of which is species diversity, interaction intensity, and system stability. A few studies demonstrated food web stability could be regarded as a signal for the state transition in a real lake ecosystem (Kuiper et al., 2015; Zhang et al., 2023). Another type is numerous ecological network analysis-based indicators. The former is clear and simple, while the latter has many controversies and has not yet formed a short list. This study mainly focuses on the indicators of the former. However, this does not mean that other indicators are not important, as they provide direction for future research.

The community-level responses of an ecosystem need to be quantified using holistic and integrated indicators. The low taxonomic resolution of groups of organisms modeled with AQUATOX prevents the use of classical taxonomy-based biological metrics (Lombardo et al., 2015). Thus, in the present study, the total biomass, interaction strength, and food web stability were selected to quantify the ecosystem response, as explained in the following.

**(1) Total biomass**

The total biomass evaluation results were selected as an assembled type indicator for use as a baseline to compare with the results for other indicators. The total biomass under BaP concentration *j* (*TB*_*j*_) is described as follows:

1 $$TB_j = \mathop {\sum}\limits_{i = 1}^n {\bar B_{i,j}}$$

where $\bar B_{i,j}$ is the average biomass for a given species *i* under BaP concentration *j* (*g*/*m*^2^ *dry*).

**(2) Interaction strength**

The interaction strength (or matrix) is defined as the effect of species *j* on the per capita growth rate of species *i* (Novak et al., 2016). Interactions between species strongly shape the structure and function of ecological communities and ecosystems (Wootton & Emmerson, 2005). In addition, ecosystem stability and functions are strongly mediated by the arrangement of the interaction strength (Berlow et al., 2004).

The interaction strength is actually a square matrix. The off-diagonal element is the interspecific interaction strength *α*_*ij*_, which represents the influence of functional group *j* on functional group *i*, such that *a*_*ij*_ < 0 and *a*_*ji*_ *>* 0 for prey *i* and predator *j* pairs. The diagonal element (*α*_*ii*_) is the intraspecific interaction strength, which is difficult to estimate accurately, and thus it is usually assumed that *α*_*ii*_ *=* 0 to avoid any bias in the evaluation of stability (Bascompte et al., 2005; Jacquet et al., 2016). According to the method used for measuring the interaction strength based on energy flow in a food web (Bascompte et al., 2005), *α*_*ij*_ and *a*_*ji*_ can be described as follows:

2 $$a_{i,j} = \frac{{ - \left( {Q/B} \right)_j\, \times \,DC_{i,j}}}{{B_i}}$$

3 $$a_{j,i} = \frac{{e_{i,j}\left( {Q/B} \right)_j\, \times \,DC_{i,j}}}{{B_i}}$$

where (*Q*/*B*)_*j*_ is the number of times predator *j* consumes its own weight per day and *e*_*i,j*_ is the efficiency of prey biomass transformation into predator biomass, with values that vary between 0 and 1. These two parameters are intrinsic constants for each species and they can be derived from the static Ecopath model of BYD Lake (Zeng et al., 2021). *DC*_*i,j*_ is the proportion of prey in the diet of the predator and *B*_*i*_ is the biomass of prey *i*. The last two parameters are obtained from the AQUATOX model of BYD Lake.

The average interaction strength ($\overline {IS}$) is employed to evaluate the systematic performance, which is described as follows:

4 $$\overline {IS} = average\left( {\left| {Matrix\left( {IS} \right)} \right|} \right)$$

where |*Matrix* (*IS*)| is the absolute value of elements in the interaction strength matrix.

**(3) Food web stability**

A Jacobian matrix *J* represents the direct effect of an individual of one species on the total population of another species at or near equilibrium (Emmerson & Yearsley, 2004). The matrix is given as follows:

5 $$J_{i,j} = \alpha _{i,j}N_i^ \ast$$

where *a*_*i,j*_ denotes elements in the interaction strength matrix (*IS*) and $N_i^ \ast$ is the biomass of species *i* near equilibrium.

The local stability of an equilibrium point can be determined by inspecting the eigenvalues of *J*. The equilibrium point is locally stable if the largest eigenvalue of *J*, *λ*_*max*_, has a negative real part (May, 1972; Pimm & Lawton, 1977):

6 $$Real\left( {\lambda _{max}} \right)\, < \,0,stable$$

otherwise,

7 $$Real\left( {\lambda _{max}} \right)\, > \,0,unstable$$

Real (λmax) come from Jacobian matrix. An element *J*_*i,j*_ in the Jacobian matrix means there is a direct effect of the average species j individual on species i’s population growth rate with all other species held constant. However, interaction strength differs in species i’s per capita growth rate (Novak et al., 2016). Formula (5) reflects the relationship between two matrices.

The related parameters used for calculating the indicators are shown in Table 1. The diet matrix is shown in Table 2.

**Table 1:** Parameters used to calculate the interaction strength and food web stability

Function Group name	(Q/B)_j_^a^ (year^−1^)	e_ij_^b^ (P/Q)_j_	B_i_ (t/km^2^)^c^						
			C_BaP_ = 0ug/L	C_BaP_ = 0.01 ug/L	C_BaP_ = 0.02 ug/L	C_BaP_ = 0.04 ug/L	C_BaP_ = 0.06 ug/L	C_BaP_ = 0.08 ug/L	C_BaP_ = 0.1 ug/L
Diatoms	/	/	0.10	0.11	0.17	0.09	0.21	0.06	0.06
Blue-Green	/	/	0.22	0.22	0.22	0.03	0.03	0.03	0.03
Myriophyllum	/	/	124.84	124.84	114.20	40.38	12.84	11.61	10.87
Rotifer	123.2	0.04	5.64	5.29	4.85	1.69	0.26	0.24	0.19
Cladoceran	270	0.09	3.13	3.13	2.63	0.95	0.20	0.04	0.02
Copepod	270	0.09	4.70	4.70	4.05	1.48	0.14	0.17	0.17
Oligochaeta	8.6	0.23	12.11	12.11	10.00	5.37	0.06	0.00	0.00
Chironomid	101	0.05	0.32	0.22	0.03	0.02	0.01	0.01	0.01
Mussel	9.75	0.31	2.87	2.87	2.88	1.77	1.00	0.86	0.72
Gastropod	9.75	0.31	14.24	14.24	14.72	8.57	3.70	2.98	2.86
Carp	10.81	0.16	75.00	75.00	46.48	30.58	20.71	19.46	18.70
Catfish	5.93	0.25	4.64	3.64	0.67	0.64	0.58	0.56	0.55
sum	/	/	247.81	246.37	200.90	91.57	39.74	36.02	34.18

^a^(*Q/B*)_*j*_ is the consumption-to-biomass ratio for species *j*. Data are from the study by Zeng et al. (2021)

^b^*e*_*ij*_ is the efficiency of prey biomass transformation into predator biomass, and the value is equal to the production-to-consumption ratio (*P/Q*). Data are from the study by Zeng et al. (2021)

^c^*B*_*i*_ is the annual mean biomass of species *i*. The values were derived from the simulation results obtained by the AQUATOX model for BYD Lake under different concentrations of BaP

**Table 2:** Food matrix for BYD Lake (t/km^2^)

Species	Diatom	Blue-Greens	Myriophyllum	Rotifer	Cladoceran	Copepod	Tubifex	Chironomid	Mussel	Gastropod	Carp	Catfish
Diatom	0.00	0.00	0.00	0.00	0.00	0.00	0.00	0.00	0.10	0.05	0.00	0.00
Blue-Greens	0.00	0.00	0.00	0.10	0.11	0.10	0.00	0.00	0.12	0.06	0.05	0.00
Myriophyllum	0.00	0.00	0.00	0.00	0.00	0.00	0.00	0.00	0.25	0.28	0.13	0.00
Rotifer	0.00	0.00	0.00	0.00	0.00	0.00	0.00	0.00	0.18	0.11	0.13	0.00
Cladoceran	0.00	0.00	0.00	0.00	0.00	0.00	0.00	0.10	0.00	0.11	0.13	0.00
Copepod	0.00	0.00	0.00	0.00	0.00	0.00	0.00	0.10	0.00	0.11	0.13	0.00
Tubifex t	0.00	0.00	0.00	0.00	0.00	0.00	0.00	0.00	0.00	0.00	0.13	0.00
Chironomid	0.00	0.00	0.00	0.00	0.00	0.00	0.00	0.00	0.00	0.00	0.13	0.00
Mussel, sensitive	0.00	0.00	0.00	0.00	0.00	0.00	0.00	0.00	0.00	0.00	0.00	0.25
Gastropod	0.00	0.00	0.00	0.00	0.00	0.00	0.00	0.00	0.00	0.00	0.00	0.25
Carp	0.00	0.00	0.00	0.00	0.00	0.00	0.00	0.00	0.00	0.00	0.00	0.26
Catfish	0.00	0.00	0.00	0.00	0.00	0.00	0.00	0.00	0.00	0.00	0.00	0.00

## Results

### Response of BYD Lake ecosystem to PAHs

**Fig. 1:**
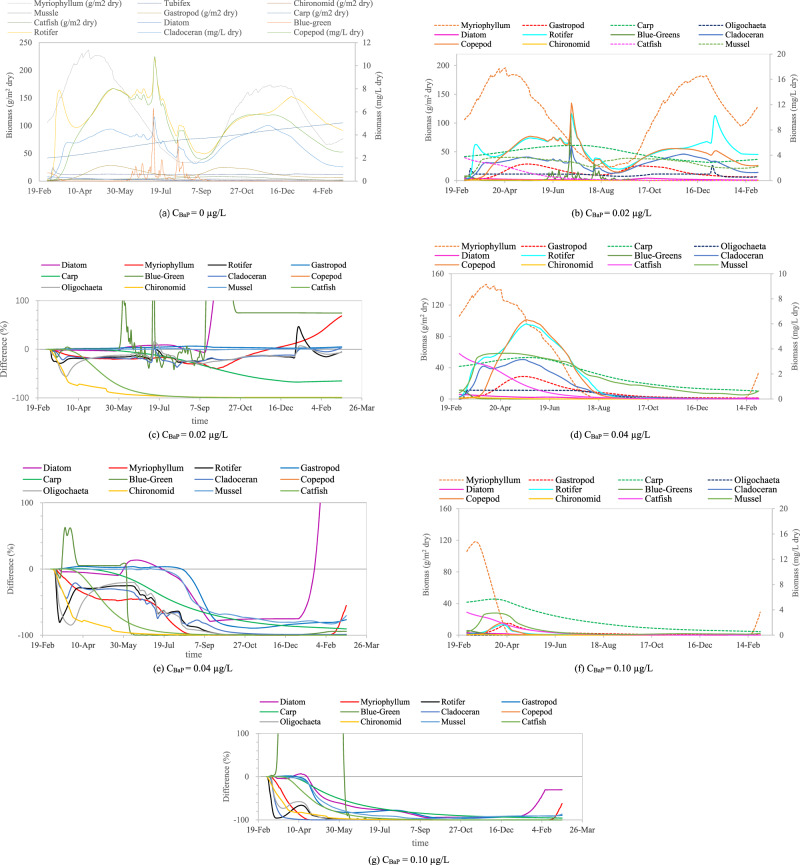
Biomass dynamics of BYD Lake community under different BaP concentrations. Note: The solid line corresponds to the left ordinate, and the dashed line corresponds to the right ordinate; **a**, **b**, **d**, and **f** show the temporal biomass dynamics of each species (simulation time from March 1, 2018 to March 1, 2019); and **c**, **e**, and **g** show the percentage of biomass change over time with the presence or absence of BaP. The percentage decrease or increase in the biomass of each species was based on the baseline of the control scenario. The total percentage of the upper limit is 1200%, because there are 12 species in total

The variations in the species biomasses over time in BYD Lake under low, medium, and high concentrations of BaP interference are shown in Fig. 1. Under the low concentration (C_BaP_ = 0.02 µg/L), the biomasses of the mussel and gastropod were basically stable from the beginning until the simulation terminated, although some fluctuations occurred. The biomasses of the other species declined initially, before following one of three patterns: increasing and then decreasing, increasing and then stabilizing, or stabilizing and then decreasing. For example, carp, catfish, and myriophyllum declined initially, before increasing and decreasing. Chironomid decreased initially, before stabilizing and then decreasing. Oligochaeta, cladocerans, copepods, and rotifers decreased initially, before increasing and then stabilizing.

Under the medium concentration (C_BaP_ = 0.04 µg/L), the increased toxicity due to BaP interference led to decreases in the biomasses of all species initially in the first stage of the simulation. The changes in the biomasses of the species in the second stage were simpler than those under the low concentration, where they all increased and then decreased. The only difference was the length of time required to decline to extinction. Thus, some opposing force delayed the toxic reaction. Under the high concentration (C_BaP_ = 0.1 µg/L), the biomasses of all species decreased to extinction or close to extinction at a faster rate. The biomasses of few species tended to increase in contrast to the changes at the low and medium concentrations.

**Fig. 2:**
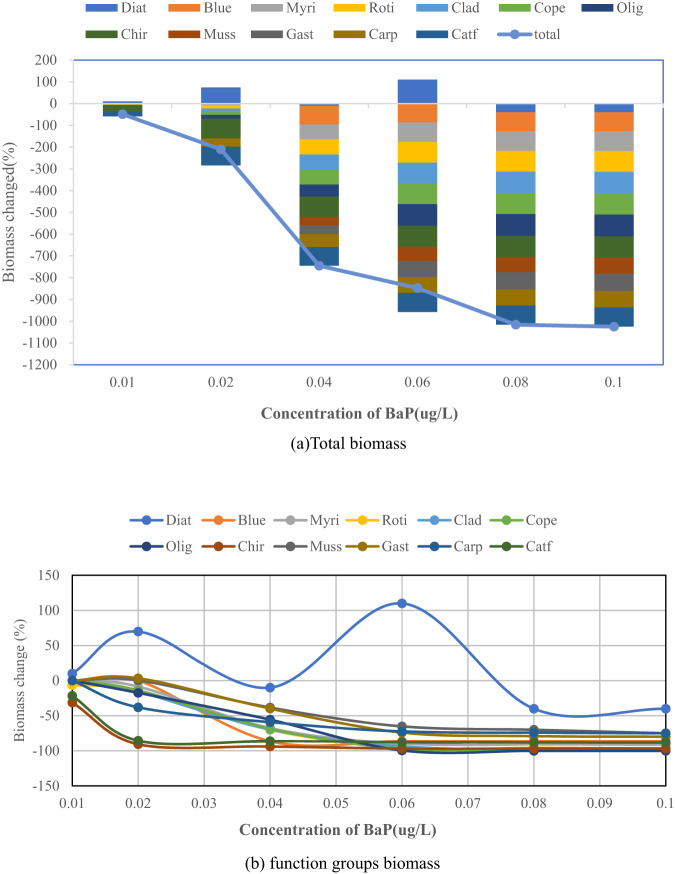
Changes in biomasses of function groups under different BaP concentrations. Diat Diatom, Blue Blue-green algae, Myri Myriophyllum, Roti Rotifer, Clad Cladoceran, Cope Copepod, Olig Oligochaeta, Chir Chironomid, Muss Mussel, Gast Gastropod, Carp Carp, Catf Catfish, total Total biomass

The averaged changes in the total species biomasses and function groups under the BaP concentration gradient are shown in Fig. 2.

As the BaP concentration increased, the biomasses of most species decreased, except the biomass of diatoms increased at low and medium concentrations (Fig. 2a). This counter-intuitive change in the biomass of diatoms can be explained by species interactions, or so-called indirect effects (Grechi et al., 2016; Zhang et al., 2018). The response in terms of the total biomass to BaP conformed to a sigmoid-shaped curve, such as a Weibull or logistic distribution. As shown in Fig. 2b, different functional groups display different response patterns. The chironomid and catfish quickly react to display S-shaped responses. Others more or less shows a hysteresis effect. The diatoms show abnormal biomass growth.

We define a significant decrease in biomass over 15% compared to the zero scenario. The species sensitivity was defined as the minimum BaP concentration where a function group was significantly affected. The smaller the minimum concentration, the more sensitive the functional group. As shown in Table 3, chironomid and catfish were the most sensitive, and their biomasses decreased significantly at a concentration of BaP = 0.02 µg/L. The next most sensitive were carp, oligochaeta, and zooplankton, where their biomasses decreased significantly at CBap = 0.04 µg/L, followed by phytoplankton and benthic animals. The least sensitive were diatoms. It shows chironomid and catfish have high sensitivity, and infers they can be employed as bioindicators of BaP pollution for BYD Lake.

**Table 3:** Biomass changes of function groups under different Bap concentrations

Function Group	Percentage reduction in biomass compared to the scenario C_Bap_ = 0 (%)					
	C_BaP_ = 0.01 ug/L	C_BaP_ = 0.02 ug/L	C_BaP_ = 0.04 ug/L	C_BaP_ = 0.06 ug/L	C_BaP_ = 0.08 ug/L	C_BaP_ = 0.1 ug/L
Diatoms	10.00	70.00	−10.00	110.00	−40.00	−40.00
Blue-Green	0.00	0.00	−86.36	−86.36	−86.36	−86.36
Myriophyllum	0.00	−8.52	−67.65	−89.71	−90.70	−91.29
Rotifer	−21.21	−24.01	−70.04	−95.39	−95.74	−96.63
Cladoceran	0.00	−15.97	−69.65	−93.61	−98.72	−99.36
Copepod	0.00	−13.83	−68.51	−97.02	−96.38	−96.38
Oligochaeta	0.00	−17.42	−55.66	−99.50	−100.00	−100.00
Chironomid	−31.25	−90.63	−93.75	−96.88	−96.88	−96.88
Mussel	0.00	0.35	−38.33	−65.16	−70.03	−74.91
Gastropod	0.00	3.37	−39.82	−74.02	−79.07	−79.92
Carp	0.00	−38.03	−59.23	−72.39	−74.05	−75.07
Catfish	−21.55	−85.56	−86.21	−87.50	−87.93	−88.15
sum	−49.01	−210.25	−745.20	−847.54	−1015.88	−1024.95

*C*_*Bap*_ Concentration of BaP in BYD Lake water

**Fig. 3:**
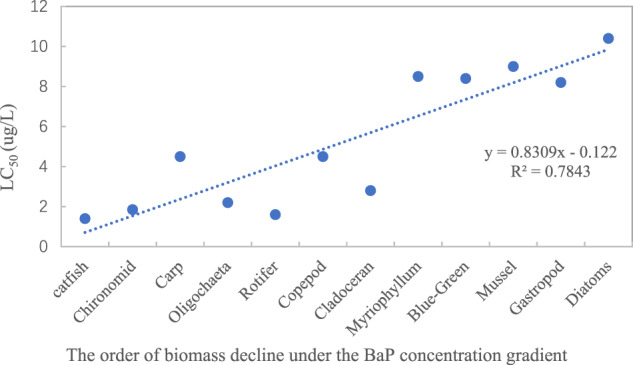
Relationships between sensitivity of species to toxicity and the LC_50_. **a** Analysis of Variance of the regression shows its *P* value is 0.0002, much Less than significance level 0.05. It infers that the regression equation is significant. **b** The order in which species appear poisoned (>15% reduction in biomass) reflects sensitivity, and the more sensitive the lower concentration of BaP cause the poisoning. At low concentration of BaP, catfish and chironomid were firstly poisoned, then zooplankton, and finally phytoplankton and benthic animals

According to the toxicokinetic process in the AQUATOX model, the sensitivity of a species to toxicity is related to LC_50_ over a short time period. When there is no interaction, the order of decline in species biomass under the BaP concentration gradient should be completely consistent with the LC_50_ order. In actual ecosystems, some species may experience hysteresis due to interactions, which were possibly inconsistent with the order of LC_50_.^.^ Figure 3 shows that the decreasing order of species biomass is basically consistent with the LC_50_ order.

**Fig. 4:**
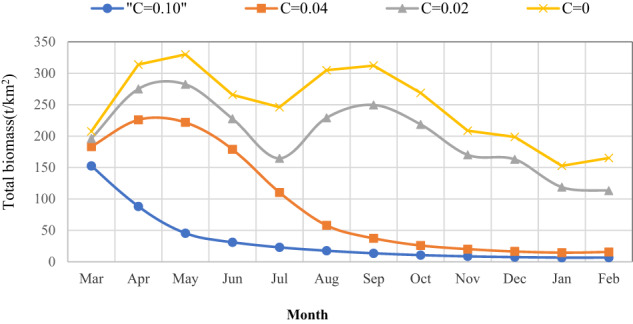
Monthly variations in total biomass under different BaP concentrations

The monthly variations in the total biomasses under different BaP concentrations are shown in Fig. 4. At a low concentration, the monthly averaged total biomass decreased and the trend was similar to that under the undisturbed scenario. However, the trend was closer to an “S” shape as the concentration increased. There is a drop in biomass in July. Perhaps due to the massive proliferation of algae in July, the competitive inhibition effect has led to a decrease in the biomass of submerged plants, leading to a decrease in the biomass of other species that rely on submerged plants as their habitat and food.

### Performance of indicators

**Fig. 5:**
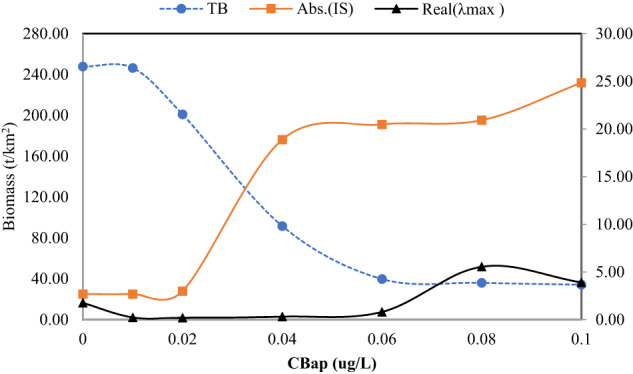
Responses of indicators under different BaP concentrations. TB total biomass (t/km^2^); Abs. (IS) the absolute mean value of the interaction strength, Real (*λ*_*max*_) the real part of the maximum eigenvalue of the Jacobian matrix, here Real (*λ*_*max*_) > 0

The results obtained for the indicators under different BaP concentrations are depicted in Fig. 5.

The decrease in the total biomass followed a sigmoid-shaped curve as the BaP concentration increased, thereby indicating that toxicity decreased the biomass. A threshold phenomenon occurred where the rate of decline increased when the threshold was exceeded. The absolute mean value of the interaction strength increased with the BaP concentration in the form of a sigmoid-shaped curve. This curve and the curve for the total biomass were symmetrical. The correspondence was good between the interaction strength and total biomass, thereby indicating that the interaction strength was effective for characterizing the ecosystem state. The increase in the interaction strength during the steady-state transition stage also suggested that it could be applied as a useful indicator of abrupt changes (or thresholds) in the ecosystem state.

The real part of the largest eigenvalue of the Jacobian matrix (Real (λmax)) was always greater than zero in this study, which indicated that the food web was unstable under different BaP concentrations. However, the stability index for the food web and the total biomass were not consistent with the curve’s shape. These findings showed that the stability index was not a good indicator of changes in the ecological state. The stability index exhibited almost no response in the steady-state transition stage when the ecological state, (e.g., total biomass and the interaction strength) was rapidly changing. Thus, the stability index may only be suitable for qualitative assessments of system dynamics, and not for quantitative analysis.

A good indicator may have the following characteristics: (1) high sensitivity. It means responses of an indicator happen at a low concentration of a pollutant. (2) properly indicating state transitions. (3) less time-leg. It refers to the less time interval from the beginning to the end of indicator changes. The total biomass has high sensitivity and properly indicating state transitions, but more time-lag. The interaction strength has less time-lag and properly indicating state transition. However, the stability indicator does not properly indicate state transition and the least sensitivity. Therefore, the total biomass and interaction strength are better than stability indicator.

## Discussion

Previous theoretical studies have explored how numerous features of ecosystems can affect stability, including the diversity (number of species), strength of interactions among species, topology of food webs, and sensitivities of species to different types of environmental perturbations (Ives & Carpenter 2007). Species are directly and indirectly connected to each other through a complex web of interactions, so impacts that affect one species in the system have ramifications on other system components through multiple direct and indirect pathways. Therefore, interaction strengths often mediate the changes in species caused by the physical and chemical environment (Wootton & Emmerson, 2005).

The toxicity of organic compounds is usually represented by toxicity end points calculated by using dose–response models (Philibert et al., 2023). Dose–response studies typically produce data that fit a sigmoid curve when the response is plotted against the dosage (Gadagkar & Call, 2015). Studies have demonstrated that the logistic and accumulative functions of Weibull’s distribution are the most suitable equations (Riobó et al., 2008). In AQUATOX, the biomass killed per day over time was computed by using a family of accumulative functions of Weibull’s distribution with different shape parameters to fit the observed cumulative fraction killed (Park & Clough, 2018). Under different shape values, the function was characterized as hyperbolic or sigmoid types. In most cases, the dose–response curve was similar to that of the total biomass shown in Fig. 2, where the proportion of deaths increased rapidly at the beginning, before increasing slowly and finally approaching the maximum death rate. In multi-species environments, species toxicity reactions occur sequentially over time in the order of the toxicity endpoint value LC_50_, as shown in Fig. 3.

**Fig. 6:**
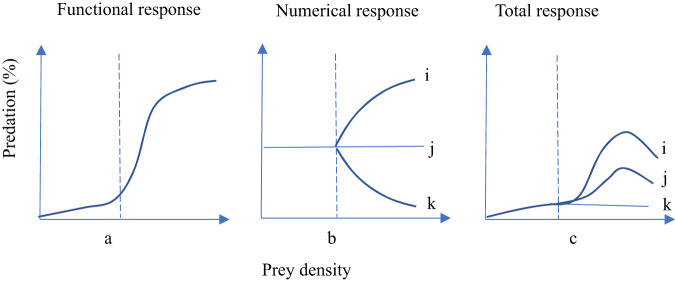
Time lagged predation types (compensatory predation). Note: i- a direct response; j- no response; k- an inverse response; Holling (1959)

Because species interaction in BYD Lake is mainly predator-prey relationship, they are represented by food matrix (Table 2). The functional response of a predator is a measure of the predator–prey interaction strength and it is used to describe the relationship between the number of prey consumed and the initial prey density (Wasserman et al., 2018). Three functional response forms have been broadly described: linear Type I, hyperbolic Type II, and sigmoidal Type III (Alexander et al., 2013). The consumption of prey depends on the densities of the predator and prey. The increase in the prey consumed per predator as the prey density increases is called the functional response. Holling (1959b) proposed that small mammal predators exhibit a functional response and found that each curve derived from field or laboratory data was characterized by an initial S-shape, before increasing to a constant maximum consumption, as shown in Fig. 6a. The change in the density of predators is called the numerical response (Holling, 1959a) and it is similar to the intraspecific interactions concept, which mainly refers to predators competing with each other as the density increases. Three possible numerical responses comprising a direct response, no response, and inverse response were proposed by Holling (1959b). However, the populations of predators cannot respond immediately to changes in the prey density, so there must be a delay in the numerical response (Holling, 1959b), as shown in Fig. 6b. Therefore, due to the functional and numerical responses, there will be a time delay in the change in the predation rate, where it increases from a very small value to a peak value, before then decreasing or remaining stable as the prey density continues to increase, as shown in Fig. 6c. In the present study, the per capita interaction strength of the predator (*a*_*i,j*_) or prey (*a*_*j,i*_) was directly proportional to the consumption rate of prey (McCann et al., 1998), and thus the time variation was characterized by different types of functional response for predators, as shown in Fig. 6c.

**Fig. 7:**
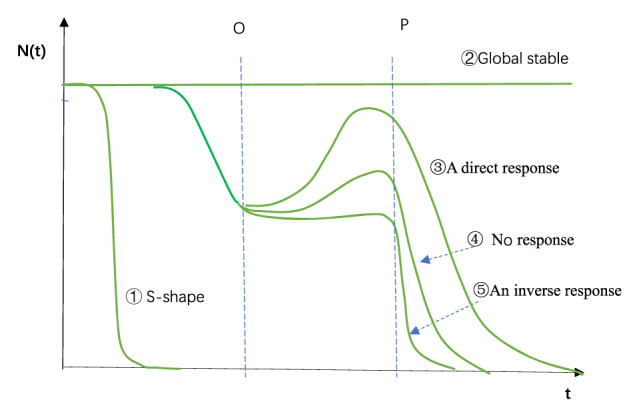
Responses of community biomass to BaP over time. Note: Observed from Figure 1c C_BaP_ = 0.02 µg/L. The ordinate is the number of the population (or population density, *N*(*t*)) and the abscissa is the time (*t*). The green lines show five possible dynamic routings of species biomass. No. ➀ is population dynamics route of highly sensitive species, such as chironomid and catfish, which shows a ‘S’-shape curved. No. ➁ is the population dynamics route of highly tolerant species. Such species (gastropod, diatom, blue-green) are not affected by toxicity at all. Nos. ➂, ➃ and ➄ are the biomass dynamics of species with intermediate sensitivity to toxicity. At first, species are more or less affected by toxicity, with an ‘S’-shaped toxicity effect in the first stage (before dashed line O). After dashed line O, the interaction strength is large enough to dominate the species biomass dynamics. After that, their possible dynamic route is ③A direct response (oligochaeta, copepod,and myriophyllum), ④ No response (cladoceran), and ⑤An inverse response (rotifer and mussel) which depend on functional and numerical responses of predation. We believe that species interactions have led to rebound after point O. The changes in predator-prey relationships among some moderately sensitive species (oligochaeta, copepod, myriophyllum, cladoceran, rotifer), resulted in an increase in biomass. After dashed line P, toxicity regain dominance and biomass declined in all species

The responses to BaP in the BYD Lake ecosystem are shown in Fig. 7.

The community dynamics of the BYD Lake ecosystem were assumed to be at equilibrium before BaP entered the lake, and the interaction strength force was equal to other inverse environmental driving forces. After BaP entered the lake, toxic effects comprised the new driving forces beyond equilibrium, with dominant roles in species dynamics. According to the sensitivity to the toxic effects of the strong, medium, and weak BaP concentrations, three types of population changes were observed comprising a strong response, medium response, and weak response, which correspond to Nos. ①, ③④⑤, and ②, respectively, shown in Fig. 7. In No. ①, a few sensitive species rapidly became extinct. In No. ③④⑤, the biomasses of other moderately sensitive species decreased from the beginning until point O, as shown in Fig. 7. After point O, the driving force affecting the population dynamics was replaced by the interaction strength, with three types of predator functional responses, as shown in Fig. 6. In No. ②, the species exhibited strong tolerance to PAH toxicity, i.e., insensitivity, and the number of species remained basically unchanged throughout the simulation period. Therefore, there were five possible changes in the population dynamics after BaP entered the lake, as shown in Fig. 7.

**Fig. 8:**
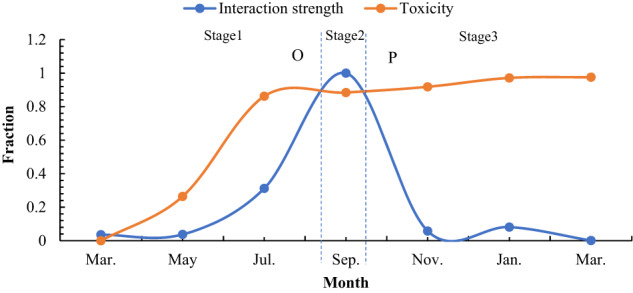
Mechanisms associated with community dynamic to BaP. The ordinate is the power of the driving force (normalized to its maximize value) and the abscissa is the time (t). Toxicity is measured with reduction rate of total biomass due to toxic reactions. However, in reality, the observed reduction is the result of a combination of toxicity and other driving forces. Therefore, we choose data at high concentration(C = 0.1ug/L) to quantify the toxicity. In this case, toxicity plays a dominant role, while other forces can be ignored. The interaction strength is calculated according to formula (2), (3), and (4), where B_i_ comes from the simulation results at a BaP equivalent concentration of C = 0.1 ug/L

The corresponding mechanisms are illustrated in Fig. 8. The community dynamics depended on the combined effects of toxicity and the interaction strength. The entire process was divided into three stages. In the first stage, the effect of toxicity increased rapidly, before reaching a maximum value and then stabilizing at this maximum value. The toxic responses of species led to changes in the species density, and thus increases in the interaction strength. However, the increases did not occur immediately due to a delay. The time difference between the loss of toxicity and interaction from equilibrium is called the time delay, and the interaction strength and toxicity intersected at point O. Toxicity was dominant before point O and the community dynamics depended on the sensitivity of each species to toxicity, with high, medium, and low response types. High response types rapidly became extinct under the influence of toxicity. Low response types appeared to be unaffected. The medium response types exhibited decreases in biomass due to certain toxic effects, but the population did not become extinct. In the second stage, the interaction strength exceeded the toxicity between point O and point P, and the interaction strength then had the major role. According to Holling’s predatory delay functional response, there are three types of responses (direct response, no response, and inverse response) between point O and point P. In the third stage, the interaction strength decreased, with a significant decrease in the community biomass due to the sustained effects of toxicity. Toxicity was dominant again after point P. The biomasses of all species decreased to extinction. In summary, the community dynamics under BaP interference depended on the competition between toxicity and the interaction strength, and the main force dominated the community dynamics process, which had different dynamic characteristics.

From the toxicological kinetic mechanism of this study, toxicity always dominates, except for a short period of time when interaction forces dominate. This implies that reducing the external loads of BaP is the top priority. When the external loads are high, the effect of using biological manipulation or bioremediation methods (Behera, et al., 2018) is short-lived. Only when the external load is close to the threshold point, combined with biological manipulation or bioremediation methods, can it accelerate community evolution and development, and restore the ecosystem. Secondly, maintaining medium to long-term biological monitoring is important. When short-term species interactions are dominant, the abnormal increase biomass in some species can interfere with our judgment, and the medium to long-term steady-state monitoring results can eliminate this interference. Finally, chironomid and catfish can be used as bioindicators for BaP pollution in BYD Lake.

Based on the predictions obtained by using the ecological model, data can be provided to help quantify the elements of the interaction strength matrix, such as the long-term series simulated species biomass, food web relationships, and food matrix. However, the quality of the parameters provided by the model depends on the model’s accuracy. It is generally considered that the ecological model is a semi-quantitative model with high uncertainty (Lombardo et al., 2015; Grechi et al., 2016), and thus the modeling results need to be interpreted very carefully. Ecological models thus typically are poorly identifiable systems, and AQUATOX is no exception. A part of the uncertainty comes from the model structure, which fails to accurately reflect the studied system. For example, undiscovered dynamic processes and simplification of the model usually cause uncertainty. The other uncertainties often related to model inputs and parameters, were derived from statistical datasets, published papers, and default parameters. The initial conditions of the system might influence the results in non-linear ecological models. Finally, data exhibit a certain level of uncertainty when model results are compared with measured data. However, Ecosystem level models represent a useful platform to assess the impact of chemicals on the structure and function of a whole ecosystem impacted by multiple stressors. The AQUATOX is probably the best-known example of this type (Lombardo et al., 2015). It has been successfully applied in chemical risk assessments. (Zhang et al., 2013, 2014, 2018; Gredelj et al., 2018). Therefore, we believed that the uncertainties of AQUATOX will not have a great influence on the core information of the research results.

Moreover, some key biophysiological parameters used to calculate the interaction strength, such as the consumption/biomass ratio and efficiency of prey biomass transformation into predator biomass, are usually empirical or analogical data, and precise experimental data support is lacking. Thus, some controlled experiments may be needed to verify the rationality of the interaction matrix element measurement method.

## Conclusions

In this study, the community responses were investigated in response to PAHs under different BaP concentrations based on an ecological model of BYD Lake. Furthermore, community indicators comprising the total biomass, interaction strength, and food web stability were compared to identify suitable indicators that accurately reflected changes in the state of the ecosystem, especially those that could reflect a steady-state transition process.

The time variation in the community response to BaP was characterized by three stages. In the first stage, the community dynamics process was dominated by toxicity with strong, medium, and weak species responses depending on the sensitivity to BaP, which was mainly determined by LC_50_. In the second stage, the dynamic process was dominated by the interaction strength, with three alternative dynamic responses comprising a direct response, no response, or inverse response. In the third stage, toxicity was dominant again. The biomasses of all species decreased to extinction. These phenomena constituted the responses of the lake communities to BaP disturbance. The toxicological dynamics at the community level were far more complex than those at the single species level, where they were influenced by the interaction strength as well as toxicity. The dynamic community toxicology process from start to finish was constantly driven by the competing effects of these two forces.

Compared with the total biomass, we found that the interaction strength index was the most suitable for use as a community-level signal. This index performed as well as the total biomass, where the toxicokinetic processes under the BaP concentration gradient followed a sigmoid-shape function, and it was a good indicator of ecosystem steady-state transitions. By contrast, the curves for the food web stability indicators did not have a sigmoid shape, and the indicators were not sensitive to changes in the ecosystem state; thus, it may only be suitable for qualitative assessments of whether the system state is stable.

This study was a preliminary attempt to identify the mechanism responsible for community dynamics driven by toxins. More theoretical analyses and case studies may be required to verify or explain the response patterns at the community level. The method used to accurately measure the interaction strength has always been an open problem. In order to acquire accurate and effective parameters, the accuracy of the ecological model needs to be improved. Thus, other methods, such as field and laboratory tests, could be used to obtain relevant data and parameters.
